# The impact of anxiety/depression symptoms on return to work in patients with chronic neck or back pain: a longitudinal cohort study from the Norwegian neck and back registry

**DOI:** 10.1186/s12891-026-10172-w

**Published:** 2026-07-09

**Authors:** Ingvild Bardal, Nils Abel Prestegård Aars, Samineh Sanatkar, Sharon A. M. Stevelink, Beate Brinchmann, Arnstein Mykletun

**Affiliations:** 1 Centre for Work and Mental Health, Nordland Hospital Trust, Bodø, Norway; 2 Department of Community Medicine, UiT, The Arctic University of Norway, Tromsø, Norway; 3 Black Dog Institute, School of Psychiatry, UNSW Sydney, Randwick, Australia; 4 Department of Psychological Medicine, Institute of Psychiatry, Psychology and Neuroscience, King’s College London, London, UK

**Keywords:** Chronic neck or back pain, Anxiety, Depression, Psychological distress, Mental disorders, Musculoskeletal disorders, Return to work, Sickness absence

## Abstract

**Background:**

Chronic musculoskeletal pain is among the leading causes of non-fatal health loss, sickness absence and disability. These patients frequently present with co-occurrent symptoms of anxiety and/or depression (A/D). Return to work (RTW) is an important measure of functional recovery, but the longitudinal impact of A/D on RTW in patients with chronic pain is less understood.

**Methods:**

This study investigates the association between A/D symptoms and RTW among sick-listed patients referred to multidisciplinary physical medicine outpatient clinics with chronic neck or back pain (*n* = 3291), using comprehensive follow-up data from Norwegian national registers. The contribution of A/D symptoms to non-RTW was calculated using population attributable fractions (PAF), with self-reported pain intensity as reference.

**Results:**

The prevalence of case level A/D symptoms was 56.8%. There was an association between case level A/D symptoms and non-RTW at 12 months both in the unadjusted model (RR 1.32, 95% CI 1.22–1.44) and after adjustment for sociodemographic factors and symptom severity (RR 1.12, 95% CI 1.03–1.23), and PAF was estimated to be 6.6% in the fully adjusted model. In a separate model with adjustment for the same variables, there were no association between pain intensity and non-RTW (RR 1.02, 95% CI 0.93–1.11).

**Conclusions:**

The results suggest that A/D symptoms are more important than pain intensity for RTW at 12 months in sick-listed patients with chronic neck or back pain, irrespective of self-reported severity of musculoskeletal symptoms and sociodemographic factors. Approximately 7% of non-RTW cases could be attributed to case level A/D symptoms. Addressing A/D symptoms as part of assessment and treatment is essential to improve RTW outcomes in patients with chronic neck or back pain.

## Introduction

Chronic pain, defined as pain lasting three months or more [1], is defined as a disease and significant public health problem [2], heavily impacting individuals, healthcare systems and workforce productivity. About 8% of U.S. adults experience severe chronic pain, limiting participation in daily activities and work [3]. Common pain locations include lower back [4] and neck [5]. These conditions, along with mental disorders, are the most prevalent for which sickness absence (SA) and disability pension (DP) are certified [4]. Many cases of chronic pain cannot be explained by underlying diseases [6] and is the leading cause of non-fatal health loss globally [4, 7, 8]. However, this has not led to adequate health policy response [9].

Chronic pain often co-occurs with mental disorders, and strong associations between anxiety, depression and chronic pain has been shown. The world mental health surveys report pooled odds ratios of 2.3 for mood disorders and 2.2 for anxiety disorders in people with- versus without chronic neck/back pain [10]. Up to 75% of patients with depression also report pain [11, 12], and nearly 1 in 20 U.S. adults live with co-occurring chronic pain and significant symptoms of anxiety and/or depression (A/D) [13]. The causal direction of this relationship is unclear; some studies suggest A/D symptoms increase chronic pain risk, while others propose the reverse [14]. Neuroimaging reveals overlapping pathways for chronic pain and depression [15], and genetic factors may explain the complex and multifactorial relationship [16].

A key goal in chronic pain treatment is helping patients regain control of their lives through functional recovery, including resuming daily activities and return to work (RTW). Employment supports quality of life by providing financial stability, purpose, social engagement and health benefits [17–19]. Conversely, inability to work worsens physical and mental health, further diminishing quality of life. Guidelines highlight RTW as a treatment goal for low back pain patients [20]. However, treating the symptoms of mental or musculoskeletal disorders alone may be insufficient to reduce SA [21].

In addition to sociodemographic and health- related predictors for SA due to pain, such as age [22], educational level [23], number of painful regions [22], fear avoidance believes [22, 24, 25], neck or back disability scores [24] and A/D symptoms [26], it has been shown that multiple factors beyond health can contribute to SA and RTW [27, 28], including work related factors [29] and disability policies [30]. Over the past decade, multidisciplinary and work-oriented interventions have been recommended as promising strategies for RTW among patients with chronic pain [31–33], although there is no consistent evidence for any work-related intervention or psychological interventions to be more efficient than others for RTW [21, 34].

Longitudinal studies examining the relationship between A/D and RTW in chronic pain patients are limited, and mechanisms for successful RTW remain unclear [33]. Understanding the factors influencing RTW is essential for improving healthcare policies and interventions for chronic pain patients.

Our study addresses this knowledge gap by exploring differences in self-reported symptom severity between sick- listed secondary care patients with chronic neck or back pain, with and without case level A/D symptoms. We examine whether A/D symptoms is associated with RTW, irrespective of musculoskeletal symptom severity, and estimate the contribution of A/D symptoms versus pain intensity to non- RTW outcomes.

## Materials and methods

We used observational longitudinal registry data from a cohort of adult patients attending multidisciplinary physical medicine outpatient clinics in Norway between 2016 and 2022. Patients were referred by general practitioners for specialist care.

Patient survey data was obtained from the Norwegian Neck and Back Registry (NNRR), a national medical quality register which routinely collects health data from patients referred to specialist health care for neck- or back pain [35]. Prior to consultation at the clinic, patients completed a questionnaire covering sociodemographic and health measures, while clinicians recorded diagnosis after the consultation. Information on SA status was retrieved from the Norwegian Labour and Welfare Administration (NAV), a national administration registry, using the patients’ social security number for linkage.

All residents of Norway are covered by the public insurance system, which provides healthcare benefits through the National Insurance Scheme, administered by NAV. When an employee is sick listed by a doctor, NAV`s workers ‘compensation program covers 100% of lost income up to 52 weeks, with employers covering the first 16 days. After one year, NAV provides long-term rehabilitation benefits such as work assessment allowance (WAA) or DP, covering about 66% of the worker`s previous income.

**Fig. 1:**
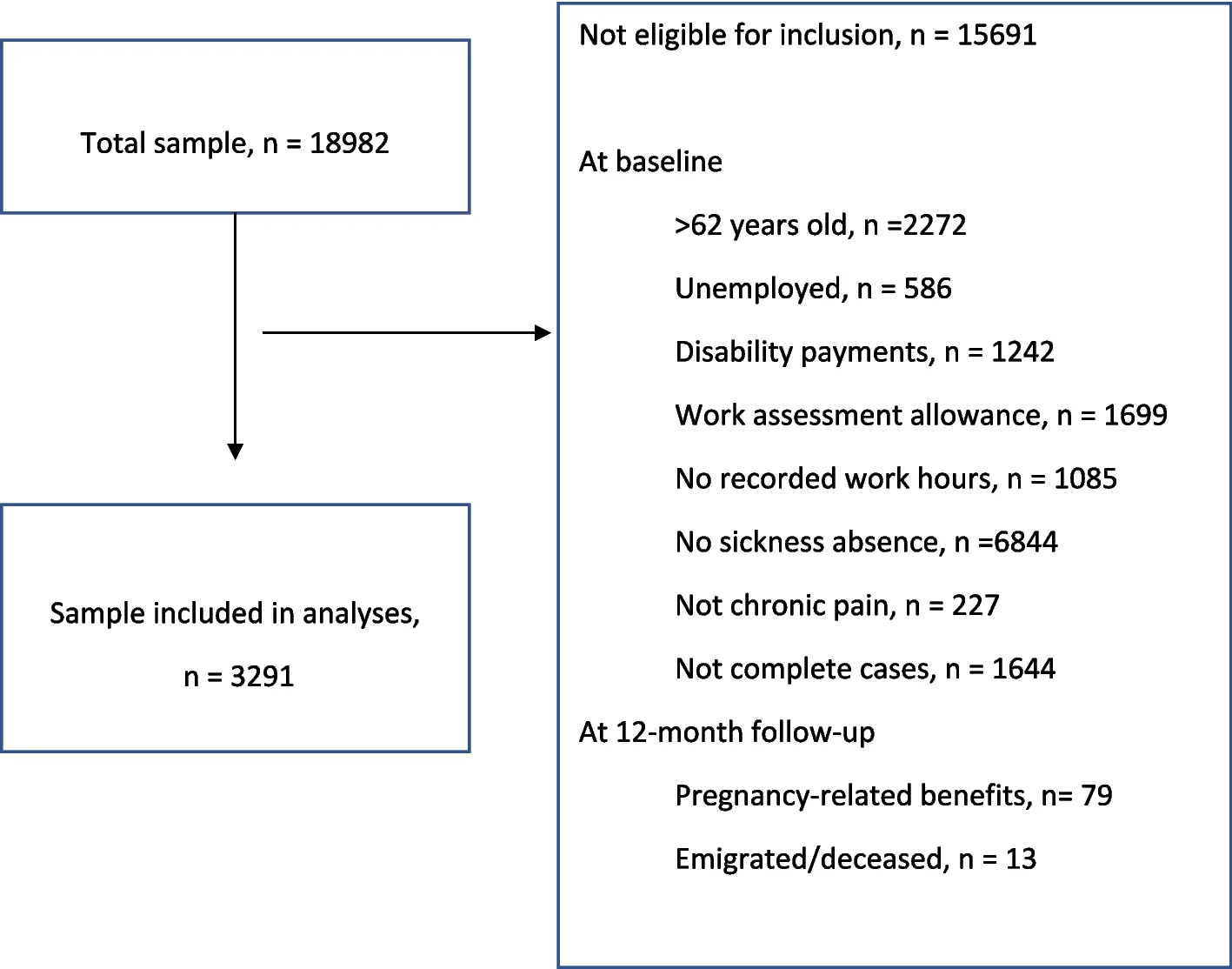
Flow diagram illustrating the inclusion process

A total of 18,982 patients were assessed for eligibility. Patients over 62 years, emigrated/deceased, unemployed, receiving long-term rehabilitation benefits (WAA or DP) receiving pregnancy- related benefits or with no working hours registered at time of assessment were excluded. The exclusion criteria were chosen based on their strong association with the outcome of non- RTW within the observation period. Finally, we also excluded patients without chronic pain, defined as pain lasting under 3 months, patients with missing data and patients currently not on SA. This resulted in a final baseline sample of 3291. A flow diagram illustrating the selection process is presented in Fig. 1.

## Variables and data measurement

### RTW status

The outcome RTW (yes/no) at 12 months after baseline was determined using NAV registry data, thus avoiding attrition as the registry is complete with no missing information. The 12-month endpoint was chosen to minimize potential confounding due to legislation on duration of SA. Non- RTW was defined as being sick listed or being recipients of long-term health benefits (WAA, DP) at follow-up. If the patient was not registered with any SA compensation, we considered the patient as working or eligible for work.

The scales used for measuring the patient’s health status were selected by the NNRR for the purpose of quality assessment of secondary care treatment for patients with musculoskeletal disorders. We included available variables based on existing literature on predictors for RTW [22–26].

### Health measures

Levels of A/D symptoms were measured by the Hopkins Symptom Checklist 10 (HSCL10), consisting of 10 items scored from 1 (not at all) to 4 (very much). The responses were summed and divided by 10 (total range: 1.0–4.0), with higher scores indicating greater symptom severity. The cut-off value of 1.85 was used to differentiate between patients with- and without case level A/D symptoms [36].

Intensity of pain during activity was measured by a numeric rating scale, reported by the patient in the range from 0 (no pain) to 10 (worst possible pain). Patients were grouped under mild to moderate pain (pain level 0–7), or severe pain (pain level 8–10) [37].

Disability was measured using either the Oswestry Disability Index (ODI) [38] for low back pain and/or the Neck Disability Index (NDI) [39] for neck pain. Each scale contains 10 items rated from 0 to 5. Total scores were converted to a percentage, where 0% indicates no disability and 100% indicates the highest disability level. In cases where both questionnaires were completed by the patient, the higher score was used in the analysis [25].

Fear-avoidance beliefs for work were measured by the Fear-Avoidance Belief Questionnaire Work (FABQ-W) [40]. Each item is rated in the range from 0 (strongly disagree) to 6 (strongly agree). The FABQ-W subscale consists of 7 items with a score range of 0–42. Higher scores indicate stronger fear-avoidance beliefs.

Further, the patient’s number of painful areas as a continuous variable was included in the analyses. Diagnosis was derived from clinicians recording and grouped into either neck- or back related health conditions.

### Sociodemographic variables

Age was included as a categorical variable in Table 1 and as a continuous variable in the analyses. Sex was reported as male or female. Education was reported in five levels, from primary school (lowest level) to university education longer than four years (highest level). Occupation was reported by patients in open questions, classified using the International Standard Classification of Occupations (ISCO-88) and reported as high-skilled white-collar occupation, low-skilled white- collar occupation, high-skilled blue- collar occupation and low-skilled blue- collar occupation [41]. For patients where a response was impossible to classify, we included a fifth category of unknown.

**Table 1:** Baseline characteristics of included patients

	Total, N = 3291
*Sociodemographic characteristics*	
Sex n (%)	
Male	1412 (42.9)
Female	1879 (57.1)
Age n (%)	
18–29 years	434 (13.2)
30–39 years	928 (28.2)
40–49 years	1012 (30.7)
50–62 years	917 (27.9)
Mean (SD)	42.3 (10.4)
Educational level n (%)	
Primary school	367 (11.2)
Vocational school	1296 (39.4)
Senior high school	406 (12.3)
University < 4 years	641 (19.5)
University ≥ 4 years	581 (17.6)
Occupation (ISCO) n (%)	
High skilled white collar	1160 (35.3)
Low skilled white collar	969 (29.4)
High skilled blue collar	414 (12.6)
Low skilled blue collar	367 (11.2)
Unknown category	381 (11.5)
*Health measures*	
Diagnosis at clinic n (%)	
Neck related condition	1650 (50.1)
Back related condition	1641 (49.9)
Number of painful regions Mean (SD)	6.7 (5.4)
Pain intensity activity n (%)	
Mild/moderate (0–7)	2087 (63.4)
Severe (8–10)	1204 (36.6)
Mean (SD)	6.6 (2.0)
ODI/NDI Mean (SD)	37.5 (14.0)
FABQ Work Mean (SD)	24.9 (9.9)
HSCL10 n (%)	
HSCL10 < 1.85	1423 (43.2)
HSCL10 ≥ 1.85	1868 (56.8)
Mean (SD)	2.04 (0.62)

Categorical variables presented as n (%) and continuous variables presented as mean (SD). SD: Standard deviation. ISCO: International Classification of Occupations. ODI: Oswestry Disability Index. NDI: Neck Disability Index. FABQ Work: Fear Avoidance Beliefs Questionnaire – Work subscale. HSCL10: Hopkins Symptom Checklist

To preserve participants’ responses and retain sample size/power, we permitted up to 25% missing data in psychometric measurements using mean imputation by the individual patient’s average on valid items. Responses on a specific scale with less than 75% completion were excluded from the analysis [42].

### Statistical analysis

Descriptive statistics were used for baseline characteristics. We compared baseline measurements of the included variables according to HSCL10 status (case level A/D symptoms vs not case level A/D symptoms) at baseline, using Chi-square tests for categorical variables and t-tests for continuous variables. Further, we examined baseline characteristics associated with RTW at 12-months follow-up.

Associations between patients` HSCL-10 scores (both as continuous and categorical variables) and RTW status 12 months after baseline were evaluated using modified Poisson regression with robust standard errors. Results are reported as Relative Risks (RR) with 95% Confidence Intervals (CI) and the corresponding p -values. We used the Poisson regression model as this enabled a calculation of population attributable fractions (PAFs) based on Levin’s formula [43]. Where case level symptoms of A/D are associated with increased risk of non-RTW (RR > 1.00), the PAF estimates the proportion of non- RTW cases that could be prevented if A/D symptoms were eliminated, assuming equal non- RTW probabilities in both groups with the remaining risk factors, and that the RR reflects a causal relationship.

In the analysis we included all covariates in five regression models: Model 1; HSCL score (unadjusted), Model 2; Model 1 and sociodemographic variables, Model 3; Model 2 and diagnoses and number of painful areas, Model 4; Model 3 and ODI/NDI and FABQ-W, and Model 5; Model 4 and pain intensity. Pain intensity was used as predictor in the same models as a separate analysis, and a z-score was computed for comparison.

Correlations between independent variables were tested with Pearson’s r for continuous variables and point-biserial correlation for binary categorical variables, and none of the variables were correlated above 0.5. Assumptions for Poisson regression was assessed and considered met. Data management and the linkage of data sources was performed using IBM SPSS Statistics for Windows, Version 29.0 (IBM Corp. Armonk, NY). Statistical analysis was conducted using Stata MP 17 (StataCorp. 2023. Stata Statistical Software: Release 17. College Station, TX: StataCorp LLC).

## Results

Table 1 presents the characteristics of the 3291 included patients. Mean HSCL score was 2.04 (SD 0.62), and 56.8% of the patients had a score of 1.85 or higher, which is estimated to represent probable case level A/D symptoms.

Table 2 shows the association between baseline characteristics and A/D symptom levels and RTW status 12 months later. Patients with case level A/D symptoms were of younger age, reported overall more severe symptoms and more painful regions than the patients without case level A/D symptoms. Overall, 57.4% of the patients had returned to work at 12 months follow-up. There were significant differences between the two RTW statuses at 12 months: patients who reported more severe symptoms, had lower education levels, or were of older age were less likely to have returned to work.

**Table 2:** Baseline characteristics associated with case level anxiety/depression symptoms and RTW at 12 months follow-up among patients with chronic pain

	Baseline			12 Months follow-up		
	HSCL10 < 1.85	HSCL10 ≥ 1.85	P for diff	RTW	Non- RTW	P for diff
	1423 (43.2)	1868 (56.8)		1888(57.4)	1403(42.6)	
*Sociodemographic characteristics*						
Sex			< 0.05			0.26
Male	646 (45.8)	766 (54.2)		826 (58.5)	586 (41.5)	
Female	777 (41.4)	1102 (58.6)		1062 (56.5)	817 (43.5)	
Age			< 0.05			< 0.05
18–29 years	147 (33.9)	287 (66.1)		277 (63.8)	157 (36.2)	
30–39 years	365 (39.3)	563 (60.7)		551 (59.4)	377 (40.6)	
40–49 years	448 (44.3)	564 (55.7)		593 (58.6)	419 (41.4)	
50–62 years	463 (50.5)	454 (49.5)		467 (50.9)	450 (49.1)	
Mean (SD)	43.6 (10.3)	41.2 (10.4)	< 0.05	41.4 (10.3)	43.4 (10.4)	< 0.05
Educational level			0.36			< 0.05
Primary school	136 (9.6)	231 (12.4)		144 (7.7)	223 (15.9)	
Vocational school	568 (39.9)	728 (39.0)		738 (39.1)	558 (39.8))	
Senior high school	178 (12.5)	228 (12.2)		238 (12.6)	168 (12.0)	
University 4 years or less	285 (20.0)	356 (19.0)		399 (21.1)	242 (17.2)	
University over 4 years	256 (18.0)	325 (17.4)		369 (19.5)	212 (15.1)	
Occupation (ISCO)			< 0.05			< 0.05
High skilled white collar	521 (44.9)	639 (55.1)		707 (60.9)	453 (39.1)	
Low skilled white collar	397 (41.0)	572 (59.0)		515 (53.1)	454 (46.9)	
High skilled blue collar	203 (49.0)	211 (51.0)		245 (59.2)	169 (40.8)	
Low skilled blue collar	158 (43.1)	209 (56.9)		187 (51.0)	180 (49.0)	
Unknown category	144 (37.8)	237 (62.2)		234 (61.4)	147 (38.6)	
*Health measures*						
Diagnosis at clinic			0.05			0.13
Neck related condition	686 (41.6)	964 (58.4)		925 (56.1)	725 (43.9)	
Back related condition	737 (44.9)	904 (55.1)		963 (41.3)	678 (58.7)	
Number of pain regions	5.6 (4.7)	7.6 (5.7)	< 0.05	6.1 (4.9)	7.6 (6.0)	< 0.05
Pain intensity activity			< 0.05			< 0.05
Mild/moderate	1024 (49.0)	1063 (51.0)		1581 (59.4)	1082 (40.6)	
Severe	399 (33.1)	805 (66.9)		305 (49.1)	316 (50.9)	
Mean (SD)	6.2 (2.0)	7.0 (1.9)	< 0.05	6.4 (2.0)	6.9 (2.0)	< 0.05
ODI/NDI	32.1 (12.4)	41.6 (13.7)	< 0.05	35.3 (13.3)	40.5 (14.3)	< 0.05
FABQ—work	22.5 (9.8)	26.7 (9.6)	< 0.05	23.3 (9.7)	27.0 (9.7)	< 0.05
HSCL10						< 0.05
HSCL10 < 1.85				910 (63.9)	513 (36.1)	
HSCL10 ≥ 1.85				978 (52.4)	890 (47.6)	
Mean (SD)				1.95 (0.59)	2.16 (0.65)	< 0.05

Categorical variables presented as N (%) and continuous variables presented as mean (SD). SD: Standard deviation. Test statistics: Independent t-test (continuous variables), Chi-squared tests of independence (categorical variables). RTW-: receiving sick leave benefit. RTW + : not receiving sick leave benefit. ISCO: International Classification of Occupations. ODI: Oswestry Disability Index. NDI: Neck Disability Index. FABQ: Fear Avoidance Beliefs Questionnaire Work subscale. HSCL10: Hopkins Symptom Checklist

Table 3 provides the results from the regression analysis. Case level A/D symptoms (HSCL10 ≥ 1.85) at baseline in the unadjusted model was associated with non- RTW at 12 months follow-up (RR 1.32, 95% CI 1.22–1.44). Adjusting for sociodemographic variables (Model 2), had little effect on the RR for non- RTW, while adjusting for diagnosis and number of painful areas (Model 3), and ODI/NDI and FABQ-W (Model 4) resulted in an RR of 1.12 (95% CI 1.03–1.23). Further adjustment for pain intensity (Model 5) did not attenuate the association. The PAF of case level A/D symptoms for non-RTW was estimated to be 6.6% in the fully adjusted model. As a comparison, the association between pain intensity and non-RTW in the same models was weaker, and the association did not remain statistically significant in the fully adjusted model (RR 1.02, 95% CI 0.93 −1.11, PAF 0.3%).

**Table 3:** Association of anxiety/depression symptoms and pain intensity with non-RTW at 12-month follow-up, N = 3291

Models (Outcome: non-RTW at 12 month)	Predictor	Continuous RR (95% CI)	p-value	Z-scored RR (95% CI)	p-value	Dichotomous RR (95% CI)	*p*-value	PAF^a^
Model 1 (unadjusted, predictor only)	A/D	1.33 (1.26–1.41)	< 0.05	1.20 (1.15–1.24)	< 0.05	1.32 (1.22–1.44)	< 0.05	15.4%
	Pain intensity	1.08 (1.06–1.10)	< 0.05	1.17 (1.12–1.22)	< 0.05	1.29 (1.19–1.39)	< 0.05	5.2%
Model 2 (Model 1 + sociodemographic factors)	A/D	1.36 (1.28–1.44)	< 0.05	1.21 (1.17–1.26)	< 0.05	1.34 (1.23–1.45)	< 0.05	16.1%
	Pain intensity	1.07 (1.05–1.09)	< 0.05	1.14 (1.10–1.19)	< 0.05	1.24 (1.15–1.34)	< 0.05	4.3%
Model 3 (Model 2 + diagnosis and painful areas)	A/D	1.31 (1.23–1.39)	< 0.05	1.18 (1.14–1.23)	< 0.05	1.27 (1.17–1.39)	< 0.05	13.5%
	Pain intensity	1.06 (1.04–1.08)	< 0.05	1.12 (1.07–1.17)	< 0.05	1.20 (1.11–1.30)	< 0.05	3.7%
Model 4 (Model 3 + ODI/NDI and FABQ-W)	A/D	1.17 (1.09–1.25)	< 0.05	1.10 (1.05–1.15)	< 0.05	1.12 (1.03–1.23)	< 0.05	6.6%
	Pain intensity	1.01 (0.99–1.03)	0.39	1.02 (0.97–1.07)	0.39	1.02 (0.93–1.11)	0.68	0.3%
Model 5 (model 4 + A/D and pain intensity)	A/D	1.17 (1.09–1.25)	< 0.05	1.10 (1.05–1.15)	< 0.05	1.12 (1.03–1.23)	< 0.05	6.6%
	Pain intensity	1.01 (0.99–1.03)	0.84	1.02 (0.93–1.11)	0.72	1.02 (0.93–1.11)	0.72	0.3%

A/D: anxiety and/or depression. Sociodemographic factors: Sex, age, educational level, occupation. ODI: Oswestry Disability Index. NDI: Neck Disability Index. FABQ: Fear Avoidance Beliefs Questionnaire Work subscale.

^a^Population Attributable Fraction (PAF) was calculated using Levins formula:$\mathrm{P}\mathrm{A}\mathrm{F}=\frac{\mathrm{P}\mathrm{e}*(\mathrm{R}\mathrm{R}-1)}{\mathrm{P}\mathrm{e}*\left(\mathrm{R}\mathrm{R}-1\right)+1}$, where Pe is the proportion of the population exposed and RR is the relative risk from Poisson regression

## Discussion

In this longitudinal observational study of RTW among sick listed patients with chronic neck or back pain, co-occurrent A/D symptoms were associated with RTW status at 12 months follow- up, both with- and without adjustments for socioeconomic factors, health measures and general symptom severity. After adjustments for sociodemographic and health factors, co-occurrent case level A/D symptoms was associated with a 12% higher risk of not returning to work, and approximately 7% of non-RTW cases could be attributed to case level A/D symptoms. In contrast, pain intensity was not associated with RTW status in the fully adjusted model.

To the best of our knowledge, this is the first study to examine how A/D symptoms is associated with RTW and the extent to which non-RTW cases can be attributed to case level A/D symptoms in a cohort of sick listed patients referred to specialist health services. By utilizing data from national registers, we provide robust evidence for the significant impact of A/D symptoms on functional outcomes among chronic neck or back pain patients.

The results confirm that co-occurrent A/D symptoms are highly prevalent among chronic pain patients and are associated with more severe symptoms across other self-reported health measures [12, 13, 44]. Consistent with previous research, we found that higher levels of pain are associated with higher levels of A/D symptoms [12, 44], and that co-occurrent symptoms of A/D negatively impacted the likelihood of RTW [26, 45]. Individuals with co-occurring A/D symptoms and chronic pain are more likely to experience functional limitations compared to those with chronic pain alone [13, 46]. Similarly, our findings further emphasize the substantial burden of A/D symptoms on functional outcomes, such as RTW. In De La Rosa et al.`s study, 69.4% of US adults with co-occurring clinically significant A/D symptoms and chronic pain reported work limitations due to a health problem, highlighting the impact of these symptoms and emphasizing the need for targeted interventions to improve functional outcomes [13]. Additionally, it has been suggested that A/D symptoms can worsen pain and pain-related disability over time [47].

Our study demonstrates that A/D symptoms are a more important predictor of RTW than pain intensity in patients referred to specialist health care treatment for chronic neck or back pain. In our unadjusted model, the suppressive effect on RTW was approximately three times higher for case level A/D symptoms (PAF 15.4%) compared to pain intensity (PAF 5.2%). In the fully adjusted model, the effect of pain intensity became negligible (PAF 0.3%), while case levels A/D symptoms still accounted for approximately 7% of non-RTW. For a common disorder like chronic pain, these findings could have a substantial clinical and public health relevance, as even small relative reductions can lead to significant improvements in RTW. The results indicate that if the case levels symptoms of A/D were eliminated, 7% of the non-RTW outcome in this population could potentially be prevented, assuming a causal relationship. Unlike other factors associated with RTW outcomes, such as educational level or age, A/D symptoms are highly modifiable in a clinical setting and thus a possible target for improved intervention.

We speculate that the contribution of A/D symptoms may be under-recognized and under-treated in a chronic pain treatment setting, as pain typically is the most prominent symptom in patients attending multidisciplinary physical medicine outpatient clinics. Historically, the emphasis on pain intensity as the primary measure of pain and pain treatment has been widespread since the introduction of the concept of pain as the “fifth vital sign” in 1996. Despite the intentions, evidence indicates that measuring pain intensity using uni-dimensional tools has not improved pain outcomes [48, 49]. Our findings support the idea that pain intensity as a self-reported measure is better understood as a personal communication about a complex and life-altering problem [49] and as a form of pain behaviour [50], rather than an isolated physical phenomenon. Measurements capturing the emotional aspects, like A/D symptoms is not only closely connected to pain intensity, but also stronger associated with functional recovery. Addressing the multifaceted nature of chronic pain, including A/D symptoms, is therefore essential, as interventions focusing solely on pain alleviation may be insufficient to achieve functional recovery.

Our findings support that multidisciplinary approaches are warranted, including elements of psychological interventions. Cognitive behavioural therapy (CBT) has shown promising results for improving RTW outcomes in patients with musculoskeletal- and/or mental disorders [51]. In a systematic review and meta-analysis, Xu et al. found that CBT-based interventions also reduced symptoms of mental illness, including depression, stress and fatigue. However, other studies have suggested that improved functioning does not necessarily correlate with reductions in pain intensity. It has been suggested that alleviation of pain is not strictly necessary for sustainable RTW, but that psychological distress may reduce the effectiveness of rehabilitation [45]. Xu`s study also highlights an important concern; while CBT interventions reduced the number of sick leave days, it did not significantly improve working ability, raising the concern of presenteeism [51]. Accordingly, the goal of functional recovery should be balanced with a focus on alleviation of symptoms.

Given that clinical guidelines for chronic pain patients emphasize work participation, our study highlights the need for further knowledge about the interaction between chronic pain and A/D symptoms, strategies to tailoring of treatment by identifying and addressing A/D symptoms, and their impact on RTW outcomes.

An important strength of the study is the use of self-reported data collected from patients in a naturalistic setting without incentives, combined with registry data for outcome measures which improves the reliability, prevents attrition and retains statistical power. Registry data are less prone to recall bias and reporting errors. Chance findings are minimized by the large sample size. However, the issue of residual confounding cannot be dismissed, as there may be unmeasured aspects that are relevant for the association. RTW might not be the treatment goal for all patients. For those with the most severe musculoskeletal conditions the treatment goal could be symptom alleviation and improved quality of life, rather than RTW. Other unmeasured aspects may include work-related factors beyond occupational category, e.g. work conditions and/or work environment, which possibly can contribute to the association between case level A/D symptoms and RTW. Generalization to other countries is limited because differences in policies can influence RTW outcomes [30]. Given the observational study design, we cannot establish causal relationships for the direction of the observed associations.

## Conclusion

Case level A/D symptoms are common among chronic neck or back pain patients and are associated with greater overall musculoskeletal symptom severity. We find that A/D symptoms are associated with RTW outcomes among sick-listed patients with chronic neck or back pain, irrespective of musculoskeletal symptom severity, diagnosis, sociodemographic factors or occupation. Co-occurrent case level A/D symptoms are associated with a 12% higher risk of not returning to work 12 months after baseline assessment, and approximately 7% of non-RTW cases can be attributed to case level A/D symptoms. In contrast, pain intensity is not an important predictor of RTW, adjusted for the same covariates. The findings suggest that addressing A/D symptoms as an integral part of treatment for patients referred to secondary care for chronic neck or back pain is both warranted and necessary to achieve functional recovery.

## Data Availability

The data supporting this article may be shared upon application to the scientific board of NNRR and NAV and requires an ethical approval from REC North.

## Abbreviations

SA: Sickness absence
DP: Disability pension
A/D: Anxiety and/or depression
RTW: Return to work
NNRR: Norwegian Neck and Back Registry
NAV: Norwegian Labour and Welfare Administration
WAA: Work assessment allowance
HSCL10: Hopkins Symptom Checklist 10
ODI: Oswestry Disability Index
NDI: Neck Disability Index
FABQ-W: Fear-Avoidance Belief Questionnaire Work
ISCO-88: International Standard Classification of Occupations
RR: Relative Risks
CI: Confidence Intervals
PAF: Population attributable fractions
